# Fifteen years of intrathecal baclofen therapy in China: complications, safety governance, and a multimodal neuromodulation strategy

**DOI:** 10.1007/s00701-026-06936-x

**Published:** 2026-06-04

**Authors:** Zhengyu Lin, Yixin Pan, Zhitong Zeng, Xiaonan Wan, Peng Huang, Dianyou Li

**Affiliations:** 1 Department of Neurosurgery, Center for Functional Neurosurgery, Ruijin Hospital Affiliated to Shanghai Jiao Tong University School of Medicine, 197 Ruijin 2 Road, Shanghai, 200025 China; 2 Department of Psychological Medicine, Institute of Psychiatry, Psychology and Neuroscience, King’s College London, 16 De Crespigny Park, Camberwell, London, SE5 8AB UK; 3 Ruijin-Hainan Hospital Affiliated to Shanghai Jiao Tong University School of Medicine, 41 Kangxiang Road, Zhongyuan Town, Qionghai, Hainan 571473 China

**Keywords:** Intrathecal baclofen, Spasticity, Case series

## Abstract

**Objective:**

To share real-world experiences and insights regarding complications associated with intrathecal baclofen (ITB) therapy from a major Chinese tertiary center, providing practical guidance for regions where ITB use is still emerging.

**Methods:**

A single-center, single-surgeon retrospective analysis was conducted on all patients who received ITB pump implantation at Ruijin Hospital (Shanghai, China) between 1st Jan 2011, and 31st Oct 2025. Data on demographics, medical history, and ITB-associated complications were extracted. Clinical effectiveness was assessed using a 5-level Likert scale of goal achievement.

**Results:**

Data from 68 individuals (44 males), representing 214.3 pump-years of therapy, were included. The most common supra-spinal pathology was cerebral palsy (10/68, 14.7%), and the predominant spinal pathology was traumatic spinal cord injury (23/68, 33.8%). 87.2% of individuals reported to have goals considered achieved or partially achieved. A total of 24 complications were documented in 19 individuals. The overall complication incidence was 0.11 events per pump-year and 0.30 per implantation. Catheter-related issues were the most frequent (8/24, 33.3%), followed by drug-related (7/24, 29.2%) and procedure-related (6/24, 25.0%) complications. Human error-related issues accounted for 25.0% (6/24) of all documented complications. Notably, refinement of surgical nuances was found to successfully mitigate wound tension and skin erosion. In addition, a multimodal neuromodulation strategy integrating sacral neuromodulation was successfully piloted in a representative case of ITB dose-limiting urinary retention.

**Conclusions:**

This study presents one of the largest reported ITB cohort in China. Our experiences underscore that long-term success in ITB therapy extends beyond surgical precision to encompass systemic clinical governance, including rigorous safety protocols and the management of refractory side effects through multimodal neuromodulation approaches. These insights, including the novel use of multimodal neuromodulation to manage dose-limiting adverse effects of ITB, may help optimize ITB practice, particularly in regions where its use is still emerging.

## Introduction

Spasticity is classically defined as a clinical symptom characterized by a velocity-dependent increase in tonic stretch reflexes with exaggerated tendon jerks, resulting from hyperexcitability of the stretch reflex [17]. It commonly arises from a variety of neurological conditions, including spinal cord injury, stroke, cerebral palsy, multiple sclerosis, and acquired brain injury [7, 17]. Spasticity can significantly interfere with mobility, functional independence, and quality of life.

For cases of severe spasticity refractory to oral medications, intrathecal baclofen (ITB) therapy can be considered to offer adequate symptom control [3, 21]. A recent meta-analysis demonstrates the efficacy of ITB in both adult and pediatric populations with various etiologies of severe spasticity [13]. Despite its efficacy, the potential for associated complications remains a critical concern, as device-, procedure-, and drug-related complications have been reported at notable rates, with many cases being life-threatening and requiring surgical revision [4, 7, 16, 18].

Although ITB therapy has been approved by the FDA since 1990 s for the treatment of severe spasticity of spinal and cerebral origin, its clinical use in China began relatively late. Consequently, there is a paucity of domestic literature on this topic [11]. Therefore, rather than providing comprehensive epidemiological data or definitive comparative outcomes, the current study aims at sharing real-world experiences and insights from a Chinese tertiary center to help bridge this gap and inform clinical practice in settings where ITB is still emerging.

## Methods

This is a single-center retrospective study. All patients who received ITB pump and catheter placement performed by single surgeon (DY. L.) at Ruijin Hospital (Shanghai, China) between the 1 st of January 2011 and the 31 st of October 2025 were considered for this study. A proportion of patients were referred from Neurology, Rehabilitation, and other related departments; however, the majority were directly evaluated through the outpatient clinic of the senior functional neurosurgeon. Patient selection for ITB implantation was based on multidisciplinary clinical assessment, including spasticity severity, refractoriness to oral therapy, and degree of functional impairment. Final treatment decisions were made after detailed counseling with the patient and caregivers regarding expected benefits, potential complications, and the need for long-term refill and follow-up adherence. Eligible patients were identified in the Hospital Information System and data pertaining patient demographics, patient medical history, and ITB-associated complications were extracted. Clinical effectiveness was assessed using a 5-level Likert scale of goal achievement to provide person-related outcomes: −2 (worse than before ITB), −1 (no meaningful change), 0 (partially achieved goal), + 1 (goal achieved), and + 2 (better than expected) [7]. To this end, individualized treatment goals were defined preoperatively through detailed shared discussions among the physicians, the patient, and caregivers, based on patient-specific needs and expectations. During this process, clinicians would guide patients toward realistic and clinically achievable expectations. The patient-reported outcome scoring was collected after dose titration was completed and clinical effect had stabilized, typically at a routine refill visit. Goal attainment was rated primarily by patient report, with caregiver input when appropriate, and documented by the treating ITB team. All participants were followed until the end of the study period. Participation ended when ITB therapy was stopped (death or explantation).

Data is presented using descriptive statistics only. For continuous variables, we report mean with standard deviation and median with interquartile range and range. For categorical variables, frequencies and percentages are presented. No inferential statistics are performed given the relatively limited number of implants and of complication events.

## Results

### Cohort characteristics and general outcomes

Participants’ characteristics are shown in Table 1. Data from 68 individuals (44 males) implanted between the 1 st of January 2011 and the 31 st of October 2025 were included, representing 214.3 years of baclofen therapy. The mean age of the patients at time of surgery was 41.4 ± 17.3 years old. The most common supra-spinal pathology was cerebral palsy (10/68, 14.7%), followed by cerebrovascular accident (8/68, 11.8%) and cerebral hypoxia (4/68, 5.9%). The predominant spinal pathology was traumatic spinal cord injury (23/68, 33.8%), followed by hereditary spastic paralysis (7/68, 10.3%) and myelopathy (3/68, 4.4%). The median dose of ITB therapy at the latest follow-up visit was 142.5 (IQR: 160.3; range: 27–420) μg/day. Three patients with spinal cord injury and refractory spasticity, initially managed with ITB monotherapy, subsequently developed persistent segmental pain that was dissociated from spasticity fluctuations. Consequently, a baclofen-morphine admixture was introduced. All three patients achieved durable pain relief without compromising antispastic efficacy.

**Table 1:** Patient characteristics at baseline and at time of latest follow-up

Characteristics	Value
Age at time of surgery (years)	
Mean (SD)	41.4 (17.3)
Median (IQR, range)	40 (28.5, 9–73)
Sex [*n* (%)]	
Male	44 (64.7%)
Female	24 (35.3%)
Etiology	
Supra-spinal [*n* (%)]	
Cerebral palsy	10 (14.7%)
Cerebrovascular accident	8 (11.8%)
Cerebral hypoxia	4 (5.9%)
Others	7 (10.3%)
Spinal [*n* (%)]	
Spinal cord injury, traumatic	23 (33.8%)
Spinal cord injury, non-traumatic	2 (2.9%)
Hereditary spastic paralysis	7 (10.3%)
Myelopathy	3 (4.4%)
Others	4 (5.9%)
Time since onset of condition (years)	
Mean (SD)	7.8 (9.0)
Median (IQR, range)	4 (8, 0.5–40)
Time since surgery (months)	
Mean (SD)	38.1 (43.5)
Median (IQR, range)	24.5 (24.8, 2–182)
ITB dosage at the latest follow-up (μg/day)	
Mean (SD)	154.4 (103.3)
Median (IQR, range)	142.5 (160.3, 27–420)

*IQR* interquartile range, *ITB* intrathecal baclofen, *SD* standard deviation

Regarding patient-related outcomes, 46.8% of individuals had goals considered achieved (score + 1), 40.4% had goals considered partially achieved (score 0), 14.9% reported no change in functional status (score −1), and 2.1% reported a worsened functional status (score −2). Main achieved goals included reduction of spasms, reduction of pain, improvement in independence for activities of daily living, and improvement in gait.

### Follow-up status and therapy discontinuation

Follow-up ended before the 31 st of the October 2025 for participants because of death (3/10, 30%), explantation for loss of effectiveness (4/10, 40%), explantation due to complication (2/10, 20%), and explantation at participant’s request (1/10, 10%). Among the four explantations for loss of effectiveness, diagnoses were hereditary spastic paraplegia (*n* = 1), hypoxic-ischemic encephalopathy (*n* = 1), and secondary dystonia after intracerebral hemorrhage (*n* = 2). Although preoperative ITB bolus trials were initially encouraging, sustained patient-reported benefit after implantation and dose optimization remained below expectations. One individual temporarily discontinued ITB therapy for financial reasons, while the pump was retained in situ, sterile water was added into the reservoir, and the infusion was programmed at the lowest flow rate.

### Characteristics of the pumps and catheters

All implanted pumps, including the primary implant and replacement, were Medtronic SYNCHROMED II (*n* = 79). All catheters implanted in our cohort were non-reinforced catheters, including model 8709, 8709SC, and 8731SC. The reinforced Ascenda models are not yet available on the Chinese market. The catheter tip was mainly located in the mid-thoracic region (between T4 and T8).

### Incidence and profile of complications

**Fig. 1:**
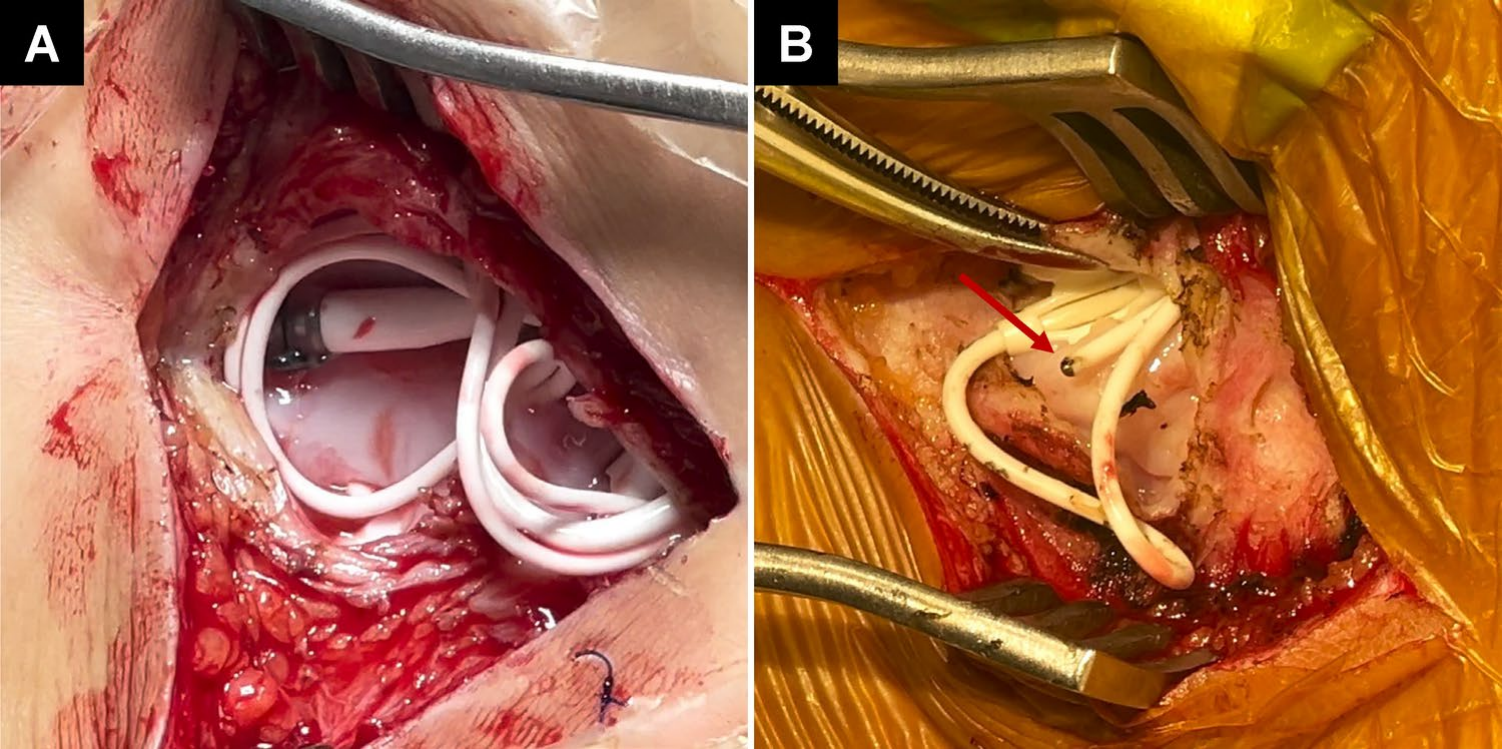
Tow cases of catheter extrusion. **A** showing the catheter displaced and coiled within the subfascial space. Red arrow in **B** indicates the tip of the catheter

**Fig. 2:**
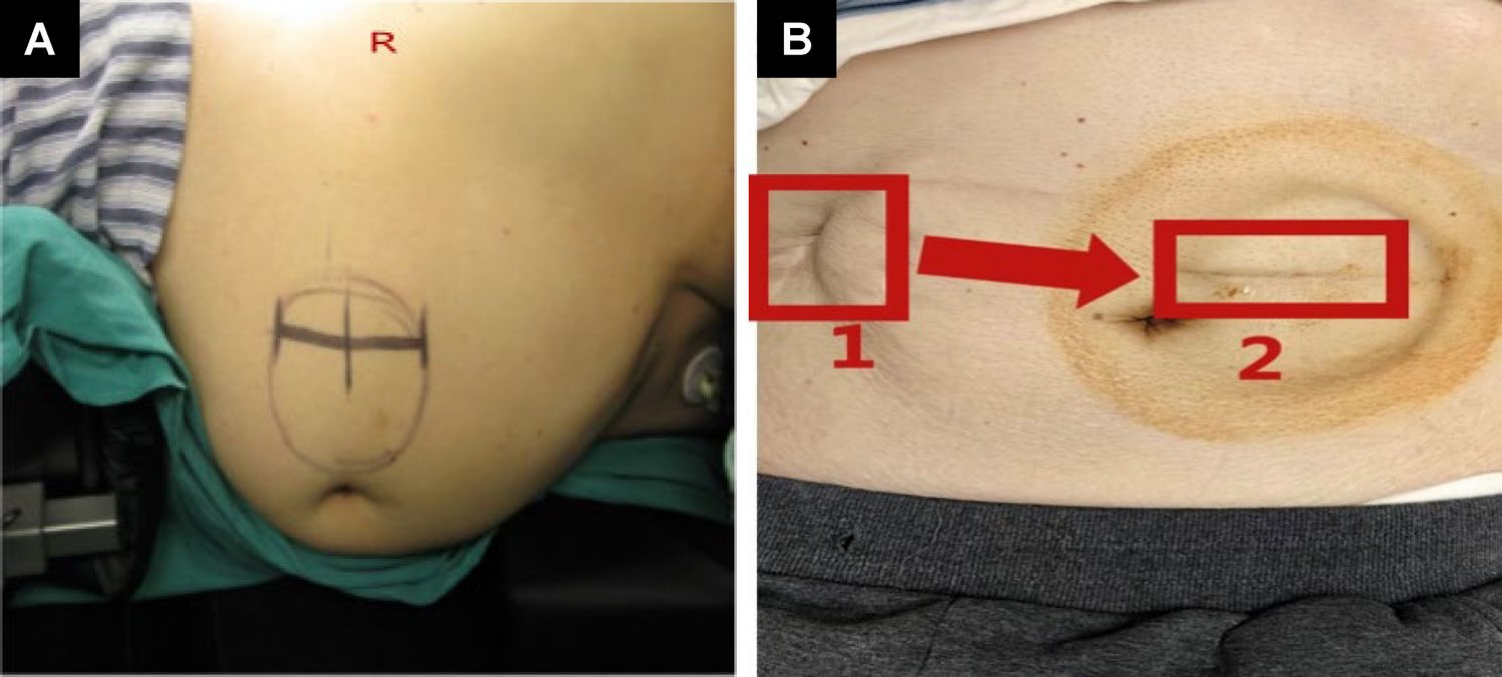
Surgical nuances: incision orientation selection for abdominal pump pocket. **A** The perpendicular incision of the primary implantation in one representative case. **B** relocation of the pump and changing the incision orientation from perpendicular to horizontal to reduce wound tension and the risk of skin erosion

During the study period, 24 complications were documented in 19 individuals; 58.3% (14/24) required hospitalization and surgical treatments (Table 2). The overall incidence of complications was 0.11 events per pump-year, and the complication rate was 0.30 per pump implantation. Catheter-related issues were the most frequent (8/24, 33.3%, see Fig. 1 for extrusion), followed by drug-related (7/24, 29.2%) and procedure-related (6/24, 25.0%, see Fig. 2 for abdominal incision orientation change to reduce the risk of skin erosion) complications.

**Table 2:** Type and rate of ITB-related complications

Complications	Value
Hardware-related	
Catheter dislocation of the distal segment	3
Catheter extrusion	2
Catheter kinking of the extra-spinal segment	1
Catheter occlusion of the distal segment	1
Catheter disconnection at the sutureless connection site	1
Pump stop due to human error	2
High residual volume probably due to human error	1
Procedure-related	
Infection/erosion	3
CSF leakage	2
Pump rotation/flipping	1
Drug-related	
Over/underdosing	3
Human error	
Refill appointment missing	2
Over/underdosing	1
Tolerance	1

Twenty-four complications were documented in 19 individuals

It is worth noting that human error accounted for 25.0% (6/24) of all documented complications. These events mainly involved programming inaccuracies during refill visits and missed refill appointments. Notably, erroneous operation of the physician controller (model 8840, Medtronic) set the pump to “stop” mode in two cases. This resulted in an immediate pump lock, triggering alarms and leading to rapid clinical deterioration due to acute baclofen withdrawal [4, 6]. Because these specific programming errors can lead to non-recoverable hardware states, both patients required unplanned, urgent surgical pump replacements to restore therapy.

### Illustrative case of ITB-sacral neuromodulation (SNM) therapeutic synergy for dose-limiting urinary retention

**Fig. 3:**
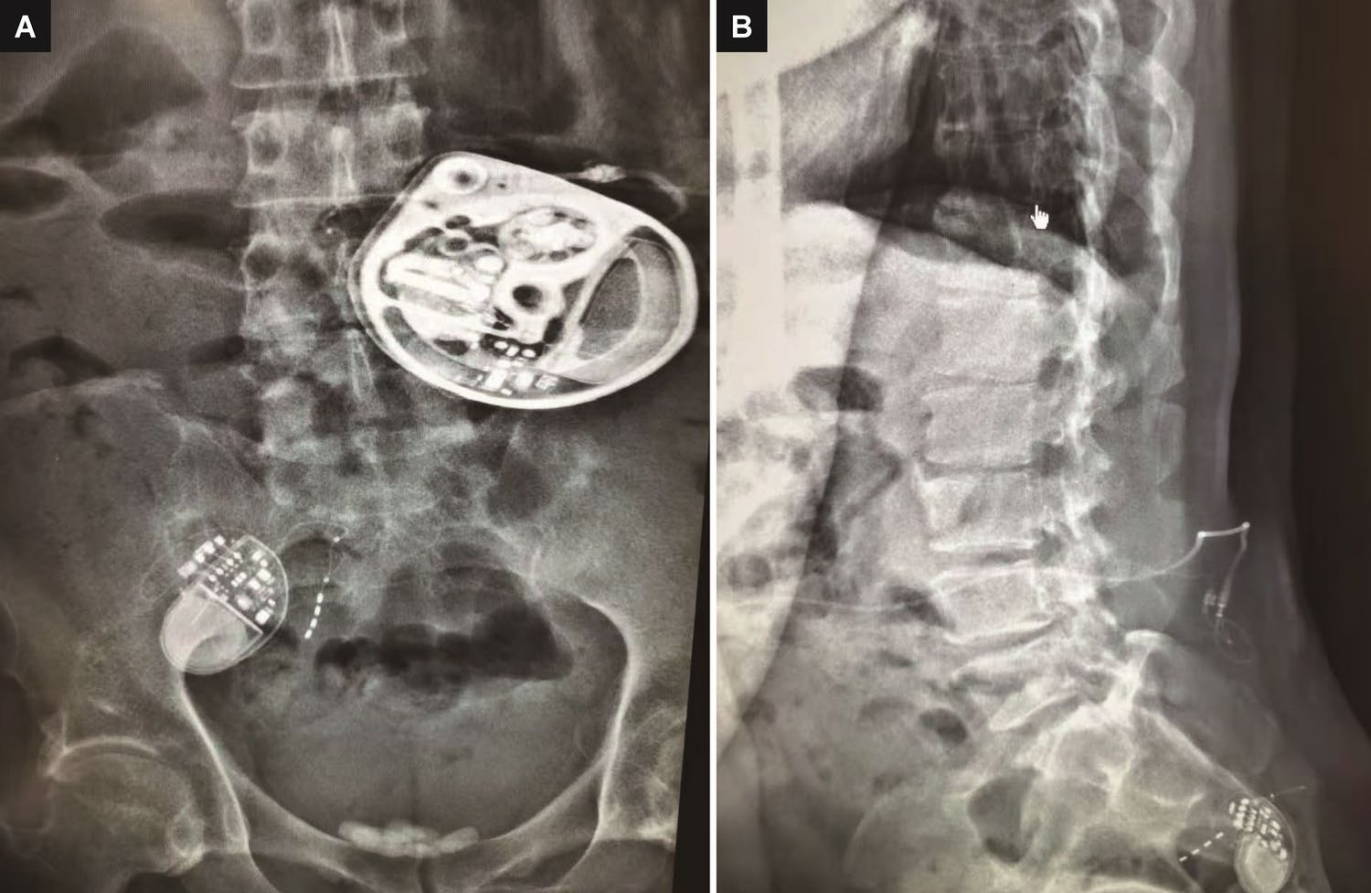
Intrathecal baclofen system combined with unilateral sacral neuromodulation. This approach improved both spasticity and troublesome urinary retention after spinal cord injury. The stimulation parameter for SNM was 2-3+, 210 μs, 14 Hz, 1.35 V. **A** antero-posterior view; **B** lateral view

In selected patients, ITB dose escalation can be limited by urinary adverse effects despite persistent need for stronger spasticity control. To preserve antispastic efficacy while addressing this dose-limiting side effect, we applied adjunct sacral neuromodulation in one representative case. A patient with thoracic spinal cord injury developed worsening urinary retention during ITB dose escalation. Rather than reducing baclofen, we integrated unilateral SNM (Fig. 3), which restored voiding function (residual urine volume reduced from 194 to 26 ml), eliminated the need for intermittent catheterization, and allowed maintenance of the target ITB dose.

## Discussion

This single-surgeon retrospective cohort represents one of the largest reported ITB series in China to date. In this cohort, most participants achieved at least partial goal attainment, and catheter-related complications were the most frequent adverse events. Our experience also highlights a potentially innovative strategy for managing dose-limiting adverse effects of ITB. To our knowledge, this is one of the first real-world demonstrations of neuromodulation synergy applied to ITB side effect management, offering a new conceptual framework for treating patients who would otherwise be considered “intolerant” to effective baclofen doses. Overall, our data underscore that long-term ITB success depends not only on surgical technique but also on structured clinical governance and individualized multimodal management.

### Incidence of complications

The incidence per pump-year found in our case series (0.11) was comparable to that reported in a recent single-center retrospective study (0.13) [7] but lower than other reports which reported an incidence of 0.2–0.3 events per pump-year [7]. Our per-implantation complication rate (0.30) also lies below the overall rate (0.41) reported in a systematic review [16]. Several factors may account for this discrepancy. Firstly, although our center is a high-volume ITB center within China (68 patients, 79 pump implantations, 214.3 years of baclofen therapy), the absolute number of implants as well as the follow-up duration remains modest on a global scale due to limited drug availability and medical insurance coverage. Furthermore, complication incidence tends to increase over time, such limited exposure could lead to fewer observed complications. Secondly, given the retrospective nature of this study and the fact that complications were retrieved and classified from medical records with a span of approximately 15 years, some drug- or procedure-related complications, especially minor events or those not requiring hospitalization, may not have been fully documented. This incomplete ascertainment may have led to underestimation of the true complication rate in this cohort.

### Safety governance and prevention of human errors

A key practical lesson from this cohort is the clinical impact of human-factor failures, especially refill-related programming errors and missed refill timing. Two cases of programming-error-induced pump stoppage were documented, resulted in acute baclofen withdrawal and urgent surgical pump replacement. A similar case has previously been reported by Mohammed I. et al. [12]. To mitigate these iatrogenic risks, we implemented an institutional refilling protocol mandating dual-clinician verification of all programming parameters. Refill procedures should be performed only by well-trained clinicians, and all drug admixtures and pump programming prescriptions must be double-checked [4]. Furthermore, both clinicians and caregivers should be aware of signs and symptoms, as well as potentially fatal consequences of over- and under-doses of ITB therapy. This approach effectively prevented recurrence of similar human errors and subsequent device-locking events throughout the remainder of the cohort.

Beyond programming inaccuracies, the omission of refill appointments represents another critical human-related risk for the discontinuation of ITB therapy. To provide a necessary safety buffer, we standardized the “low reservoir volume” alarm threshold to at least 1.0 ml. This low-reservoir threshold in our protocol was primarily informed by manufacturer guidance, as pump delivery accuracy may decline when reservoir volume falls below this value. We acknowledge that the default low-reservoir threshold by the manufacturer is 2.0 ml, which provides a wider safety margin. In our practice, this is balanced against patient financial burden. Higher thresholds may be applied in selected high-risk situations (e.g., patients with high daily dosage, history of withdrawal events, long-distance travel, lack of caregiver support, etc.). Furthermore, we implemented a scheduling strategy where refill appointments are ideally set one week prior to the calculated alarm date.

### Hardware-related complications

Catheter-related events remained the predominant complication category, consistent with previous reports [7, 16]. The catheter type, specifically non-reinforced versus reinforced catheters, has been identified as an independent risk factor for catheter-related complications in ITB therapy [2, 7, 14, 18]. However, reinforced catheters are not currently available in China, thus only non‑reinforced catheters are in use in this cohort. Among our patients, we documented one event of catheter occlusion probably due to hyperproteinorachia and high CSF viscosity. We proposed that lower drug concentration, higher infusion rate, and “Flex” infusion mode with daily boluses may be beneficial to prevent catheter tip occlusion. Therefore, in some patients it is necessary to carefully balance refill intervals, efficacy, and risk of catheter occlusion.

Besides hyperproteinorrachia, the current literature has reported other factors that may contribute to catheter occlusion or malfunction, including: (1) long-term high-dose infusion triggering inflammatory process and fibrotic scar tissue formation [10]; (2) suboptimal anchoring location or tip position in relation to vertebra leading to bending, kinking or compression [8]; (3) material fatigue over long-term use impairing catheter integrity [5], etc. The introduction of the reinforced catheters has been reported to significantly lower the catheter-related complications in ITB therapy [5, 7, 18].

The two cases of catheter extrusion (Fig. 1) were possibly due to frequent supine sit‑ups within 3 months after surgery. Indeed, greater independence in activities of daily life was found to be associated with higher incidence of catheter-related complications based on mechanical stress [7, 14, 18]. Therefore, both patients at higher risk and their caregivers should be thoroughly informed preoperatively, and any aspects of physical rehabilitation that might affect the ITB system should be discussed and managed collaboratively.

### Procedure-related complications

The incision of the abdominal pocket and volume seem to be critical to avoid potential cutaneous and catheter-pump junction issues. In our cohort, a perpendicular incision was considered as a key factor that likely contributed to skin erosion and implant exposure as it increased tension at the wound site and subsequently the incisional scar might be more fragile to friction against the skin (Fig. 2). Moreover, the size of the pocket must appropriately match the pump. An excessively large space may lead to pump hypermobility, increasing the risk of pump rotation or flipping and thereby causing catheter‑related complications; on the other hand, a pocket that is too small may impose excessive tension on the incision [1]. Another clinical vignette is that orienting the apex of the pump toward the lower inner abdomen helps minimize discomfort caused by contact with bony structures during movements and makes it easier to identify pump rotation.

### Drug-related complications

Over- or under-dosing constituted the most common drug-related complications in our case series, which necessitated outpatient reprogramming. From our impression, over- and under-dosing seem to occur more frequently in patients for whom retaining some muscle tone is important to preserve mobility. These patients may therefore require a lower drug concentration and more finely tuned programming.

One event of tolerance (1.5%) was documented in our cohort. Tolerance is defined as an escalation of the dose required to produce a previously obtained effect or by the decrement of the effect produced by a given dose of drug [9]. The diagnosis of pharmacological tolerance should be made only after ruling out any hardware-related failure of ITB system. Pulsatile bolus infusion and drug holidays are both effective in reducing the daily baclofen dose. However, it should be noted that during any drug‑holiday, alternative muscle relaxants (e.g., clonazepam) should be administered to reduce the risk of withdrawal syndrome and mitigate rebound spasticity.

Transient worsening of spasticity was managed by first identifying and promptly treating reversible precipitating factors, while temporary bridging measures such as short-term oral baclofen, with clonazepam when needed, were used for symptom control. In patients with access to a patient programmer, temporary bolus could be delivered under clinical supervision. Definitive ITB dose reprogramming was performed after reassessment following trigger resolution.

### Therapeutic synergy: Multimodal neuromodulation in managing ITB-related urinary retention

A persistent clinical dilemma in ITB therapy is the trade-off between achieving optimal spasticity control and managing bothersome drug-related side effects. In our cohort, urinary retention emerged as a significant limiting factor during dose titration, particularly in patients with spinal cord injury. Traditionally, such side effects necessitated a reduction in the baclofen dose, which frequently led to suboptimal antispastic efficacy.

Our experience suggests that these side effects do not always preclude the continuation of effective ITB therapy. Instead, a “therapeutic synergy” approach, combining ITB with complementary SNM, may help expand the therapeutic window. This synergistic paradigm offers a promising pathway for managing complex patients who would otherwise be considered “intolerant” to effective doses of ITB. Indeed, SNM has been investigated in selected patients with neurogenic lower urinary tract dysfunction, including incomplete spinal cord injury, with encouraging outcomes across mainly observational studies [20]. Prior studies also support that unilateral SNM would be sufficient to improve urinary voiding outcomes in many cases [15, 19]. In our cohort, however, this combined ITB + SNM strategy was applied in a single case and should therefore be interpreted as hypothesis-generating pending prospective validation.

### Limitations

This study has several limitations that should be considered. Methodologically, the retrospective and descriptive nature of this work precludes robust causal inference or statistical comparison with established international cohorts. It also limited our ability to identify independent risk factors or perform meaningful subgroup comparisons. Regarding data quality and follow-up duration, the retrospective documentation may have underrepresented minor complications, particularly those managed in outpatient settings. This potential underestimation is compounded by the variable follow-up periods, which may have hindered the detection of late-onset events such as catheter fatigue or pump erosion. The generalizability of our findings is also constrained by the single-center, single-surgeon setting and specific regional factors. Notably, device-availability constraints in China (specifically the exclusive use of non-reinforced catheters), as well as access-related constraints (specifically an underdeveloped referral system, limited reimbursement coverage, and limited availability of intrathecal baclofen formulation) may introduce bias compared with international cohorts. Additionally, the cohort composition reflects local practice patterns, characterized by a high proportion of traumatic spinal cord injury and limited pediatric representation, which may differ from Western or multicenter populations. Finally, several practice insights reported here, including the novel use of multimodal neuromodulation to manage ITB-related side effects, should be interpreted as hypothesis-generating rather than definitive, and warrants prospective validation.

## Conclusion

To conclude, in addition to presenting the largest retrospective cohort to date of patients receiving ITB therapy in China, the true value of this 15-year experience resides in the progressive evolution and refinement of our management paradigms. These advances encompass the establishment of rigorous safety protocols, the optimization of surgical nuances, and the integration of multimodal neuromodulation approaches to address refractory drug-related side effects. Our experience suggests that integrating complementary neuromodulation modalities, such as sacral neuromodulation, may overcome dose limiting side effects and extend the durability of ITB therapy. Collectively, these accumulated “lessons learned” provide a structured and practical roadmap for improving patient safety and extending therapy durability in clinical settings where ITB is still emerging.

## Data Availability

The datasets generated and analyzed during the current study are not publicly available due to ethical restrictions but are available from the corresponding author on reasonable request.
